# Developments and future prospects of personalized medicine in head and neck squamous cell carcinoma diagnoses and treatments

**DOI:** 10.1002/cnr2.2045

**Published:** 2024-03-24

**Authors:** Shalindu Malshan Jayawickrama, Piyumi Madhushani Ranaweera, Ratupaskatiye Gedara Gunaratnege Roshan Pradeep, Yovanthi Anurangi Jayasinghe, Kalpani Senevirathna, Abdul Jabbar Hilmi, Rajapakse Mudiyanselage Gamini Rajapakse, Kehinde Kazeem Kanmodi, Ruwan Duminda Jayasinghe

**Affiliations:** ^1^ Centre for Research in Oral Cancer, Faculty of Dental Sciences University of Peradeniya Kandy Sri Lanka; ^2^ Faculty of Information Technology, Horizon Campus Colombo Sri Lanka; ^3^ Oncology and Radiotherapy Unit National Hospital Kandy Sri Lanka; ^4^ Department of Chemistry, Faculty of Sciences University of Peradeniya Kandy Sri Lanka; ^5^ School of Dentistry University of Rwanda Kigali Rwanda; ^6^ Faculty of Dentistry University of Puthisastra Phnom Penh Cambodia; ^7^ Cephas Health Research Initiative Inc Ibadan Nigeria; ^8^ School of Health and Life Sciences Teesside University Middlesbrough UK; ^9^ Department of Oral Medicine and Periodontology, Faculty of Dental Sciences University of Peradeniya Kandy Sri Lanka

**Keywords:** artificial intelligence, biomarker, genetic data, head and neck squamous cell carcinoma, machine learning, personalized medicine, targeted therapy

## Abstract

**Background:**

Precision healthcare has entered a new era because of the developments in personalized medicine, especially in the diagnosis and treatment of head and neck squamous cell carcinoma (HNSCC). This paper explores the dynamic landscape of personalized medicine as applied to HNSCC, encompassing both current developments and future prospects.

**Recent Findings:**

The integration of personalized medicine strategies into HNSCC diagnosis is driven by the utilization of genetic data and biomarkers. Epigenetic biomarkers, which reflect modifications to DNA that can influence gene expression, have emerged as valuable indicators for early detection and risk assessment. Treatment approaches within the personalized medicine framework are equally promising. Immunotherapy, gene silencing, and editing techniques, including RNA interference and CRISPR/Cas9, offer innovative means to modulate gene expression and correct genetic aberrations driving HNSCC. The integration of stem cell research with personalized medicine presents opportunities for tailored regenerative approaches. The synergy between personalized medicine and technological advancements is exemplified by artificial intelligence (AI) and machine learning (ML) applications. These tools empower clinicians to analyze vast datasets, predict patient responses, and optimize treatment strategies with unprecedented accuracy.

**Conclusion:**

The developments and prospects of personalized medicine in HNSCC diagnosis and treatment offer a transformative approach to managing this complex malignancy. By harnessing genetic insights, biomarkers, immunotherapy, gene editing, stem cell therapies, and advanced technologies like AI and ML, personalized medicine holds the key to enhancing patient outcomes and ushering in a new era of precision oncology.

## 1 INTRODUCTION

**FIGURE 1:**
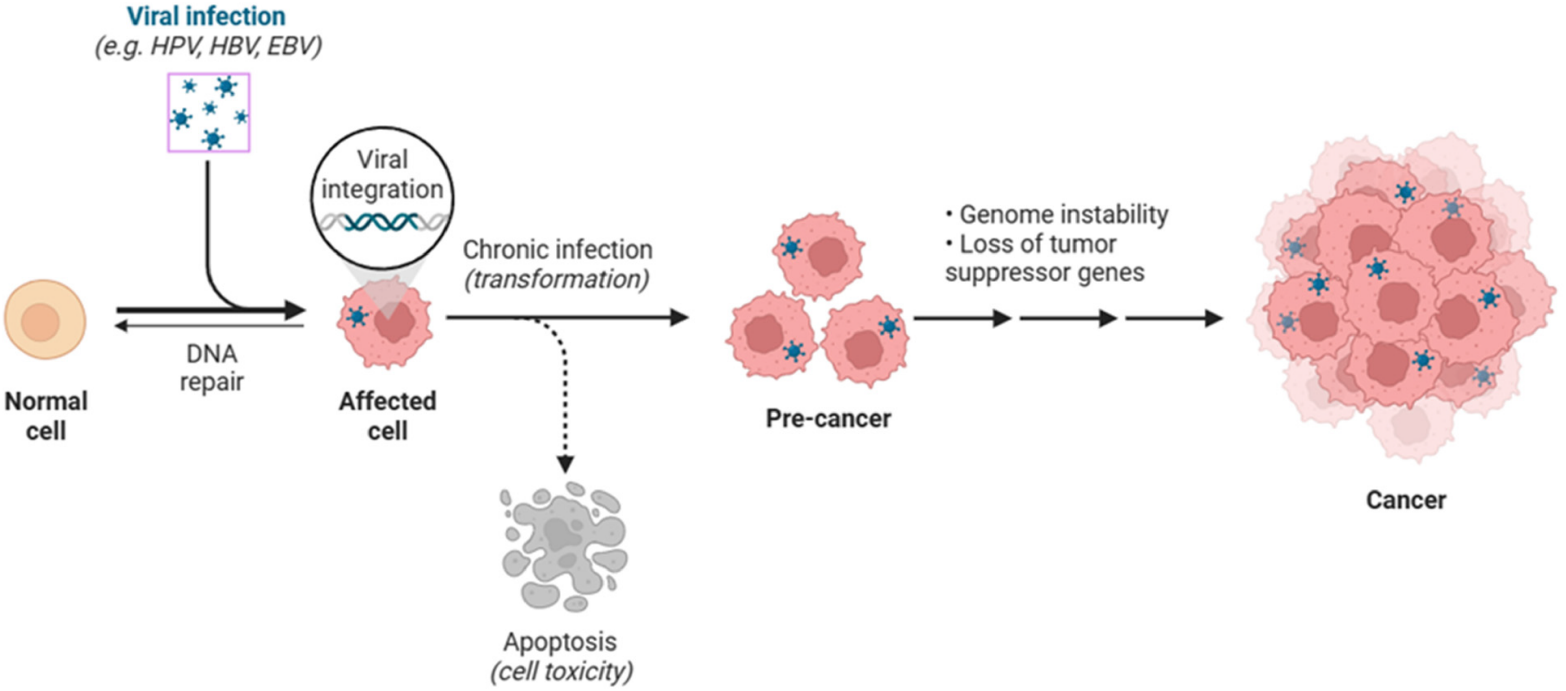
Schematic representation of the process of carcinogenesis as a result of HPV integration into the cell genome.

Cancers which occur in the squamous cells, lining mucosal surfaces of the head and neck are collectively known as head and neck squamous cell carcinomas (HNSCC).^1^ HNSCC includes malignancies that affect the salivary glands, nasal and oral cavities, larynx, pharynx, hypopharynx, and paranasal sinuses, thus cancers that arise in the eyes, esophagus, brain, and thyroid glands, and the skin of the head and neck region are excluded as HNSCC.^2^, ^3^ According to the most recent Global Cancer Observatory (GLOBOCAN) data published in 2020, stated HNSCC is the seventh among the highest prevalent cancers worldwide, which amounts to about 890 000 (∼4.5%) expected new cases and 450 000 (∼4.6%) deaths per annum.^4^ India, is recorded as the country with the greatest incidence rates in Southeast Asia, where ∼80% of all instances of HNSCC are associated with tobacco consumption along with areca nut.^5^ Incidence of HNSCC has recently grown in several nations, particularly in younger people, with an estimation of 30% of annual increase by 2030.^4^ Part of this tendency is ascribed to lifestyle alterations, such as tobacco and alcohol usage. Apart from that, in most countries, human papillomavirus (HPV) is linked to oropharyngeal cancer.^6^ Infection with HPV increases the chances of development of HNSCC.^7^ HNSCCs with HPV positivity are well‐differentiated, develop early, and have a favorable prognosis, when compared to that of HPV‐negative HNSCCs.^7^ Although there is diversity in the pathogenicity of cervical cancer (Figure 1), nothing is known about the relevance of HPV strains to HNSCC.^8^ According to projections, HPV will surpass tobacco as the primary cause of HNSCC cancer worldwide.^7^

The standard diagnostic methods for HNSCC include a detailed medical history and thorough physical examination. Performance of endoscopy, laryngoscopy, and biopsy is the invasive diagnostic procedures used traditionally.^9^, ^10^ At present, with the advancement of technology, apart from these invasive methods non‐invasive techniques such as computed tomography (CT) scan, positron emission tomography (PET), and magnetic resonance imaging (MRI) are followed as an early detection of HNSCC.^11^, ^12^

Surgery, chemotherapy, and radiation therapy are the presently used conventional methods in HNSCC treatment. However, these methods have serious adverse effects, impacting on the daily lifestyle/standards of living of patients. Acute and prolonged complications which arise due to radiation therapy in HNSCC result in sudden oral health deteriorations.^13^, ^14^

## 2 PERSONALIZED MEDICINE

A medical concept termed “personalized medicine” (PM), often referred to as precision medicine (PM), divides patients into various groups according to their expected response or risk of contracting a disease. This method customizes medical choices, procedures, interventions, and/or goods to the level of each patient individually.^15^ Although these two expressions are individually used to describe specific meanings, the terms P4 medicine, PM, personalized medicine, and stratified medicine could be used interchangeably to describe the concept.^16^ Although it has been standard practice to treat each patient individually since Hippocrates, the emergence of new diagnostic and informatics tools that aid in understanding the molecular basis of disease, particularly genomics, has made this concept gain more popularity at current.^17^ This provides us with a clear basis on which to group or stratify patients who are linked. personalized medicine has been cited as one of the 14 Grand Challenges for Engineering, an initiative supported by the National Academy of Engineering (NAE), as a critical and promising strategy to “achieve optimal individual health decisions,” thus resolving the challenge in “Engineer better medicines.”^18^, ^19^

Diagnostic testing is frequently used in PM to select the best therapies for a patient based on their genetic makeup or other molecular or cellular analyses.^20^ Initially used to genetics, the phrase “personalized medicine” has subsequently come to refer to other kinds of personalization strategies, such as the use of proteomics imaging analysis and nanoparticle‐based theranostics.^21^, ^22^

PM encourages customizing healthcare by adapting medical decisions, procedures, or products to a particular patient population as opposed to taking a one‐drug‐fits‐all strategy. Diagnostic testing is widely used in PM to select the optimal medications for a patient based on their genetic makeup or other molecular or cellular analyses. Molecular diagnostics, imaging, and analytics may be used as PM tools.^23^

HNSCC is a complex disease with clinical and biological heterogeneity. personalized medicine is an approach that aims to tailor treatment to the individual patient based on the unique characteristics of their disease.^24^ The clinical and molecular heterogeneity of HNSCC is not well addressed by the present therapy paradigm.^25^ Data from HNSCC genomic profiling studies have identified genetic characteristics specific to certain subgroups of the disease. As a result, targeted therapies have been created that can be used to treat patients with specific molecular alterations.^26^ This review aims at the advancements of personalized medicine in diagnoses and treatments in HNSCC by combatting the current issues in this regard.

## 3 DIAGNOSIS IN PERSONALIZED MEDICINE

Rapidly advancing field of genomics and genetic data analysis has brought new hope to the realm of oncology by offering a revolutionary approach to cancer treatment. Consequently, individuals with the same type of cancer may require different treatments depending on the unique genomic characteristics of their tumors. As our understanding of the genome and genetic data has been advanced and targeted drugs have been developed, genomic biomarker testing has become an essential aspect of medical care. This has led to a significant rise in the number of patients undergoing genomic tumor sequencing, and the scope of genes routinely sequenced has expanded rapidly.^27^, ^28^, ^29^

### 3.1 Genetic testing

Infection with HPV is now recognized as a known risk factor for HNSCC, especially oropharyngeal carcinoma, using genetic testing. HPV‐related diagnoses showed detectable ctHPV16DNA.^30^ Using this genetic data can predict HPV‐positive tumors often have a better prognosis and may respond differently to treatment compared to HPV‐negative tumors. Epidermal growth factor receptor (EGFR) mutations are also common in head and HNSCC.^31^ According to Okuyama et al., HPV infection has the potential to influence the expression of TMEM16A(11q13 gene amplification), in addition to affecting the EGFR, which has been identified as a possible co‐biomarker for HPV‐positive cancers due to its phosphorylation. Given the functional association between EGFR and TMEM16A and their role as co‐biomarkers for HPV, there could be interplay or crosstalk between TMEM16A expression and the development of HPV‐induced HNSCC.^32^ In cases of HNSCC with EGFR mutations, some patients may develop resistance to EGFR tyrosine kinase inhibitors (TKIs) over time. Genetic testing can identify the specific mutations responsible for resistance, guiding the selection of alternative treatment options. Similar to breast cancer,^33^ HER2 overexpression can also occur in HNSCC. Thus, determining the HER2 status of the tumor can assist in locating patients who could get benefitted from HER2‐targeted therapies.^34^ Genetic testing can assess the level of PD‐L1 expression in tumor cells, where vaccines, together with therapies focusing on the PD‐1 and TIGIT signaling pathways can help predict the potential response to immunotherapy.^35^ Some HNSCC may have mutations in DNA repair genes like BRCA1 or BRCA2.^36^ Identifying these mutations can have implications for treatment, as they may respond differently to certain therapies, such as PARP inhibitors.^37^ The chance of cancer recurrence may be increased by specific genetic markers. Identifying these markers through genetic testing can help oncologists determine appropriate surveillance and follow‐up plans for patients and genetic testing can identify HNSCC patients who have specific genetic characteristics that match the inclusion criteria for clinical trials evaluating targeted therapies or innovative treatment approaches.

More research is needed to better understand how differences in ion distribution within and outside cells impact the expressions of EGFR, PD‐L1, and TMEM16A. This understanding is crucial in deciphering how it influences the formation and operation of the PD‐1/PD‐L1 axis. Additionally, exploring the levels of EGFR, PD‐L1, and TMEM16A in early‐stage HNSCC is vital to confirm the viability of the targeted therapy proposed in this review for use in the early stages of this disease.

### 3.2 Epigenetic biomarkers

Epigenetic biomarkers are specific molecular modifications or patterns in the epigenome (the chemical modifications of DNA and associated proteins) that can provide information on the incidence, progression, prognosis, or treatment response of a particular disease, such as cancer. These biomarkers are often used for diagnostic, prognostic, or predictive purposes and can help guide medical decisions and treatment strategies.

**FIGURE 2:**
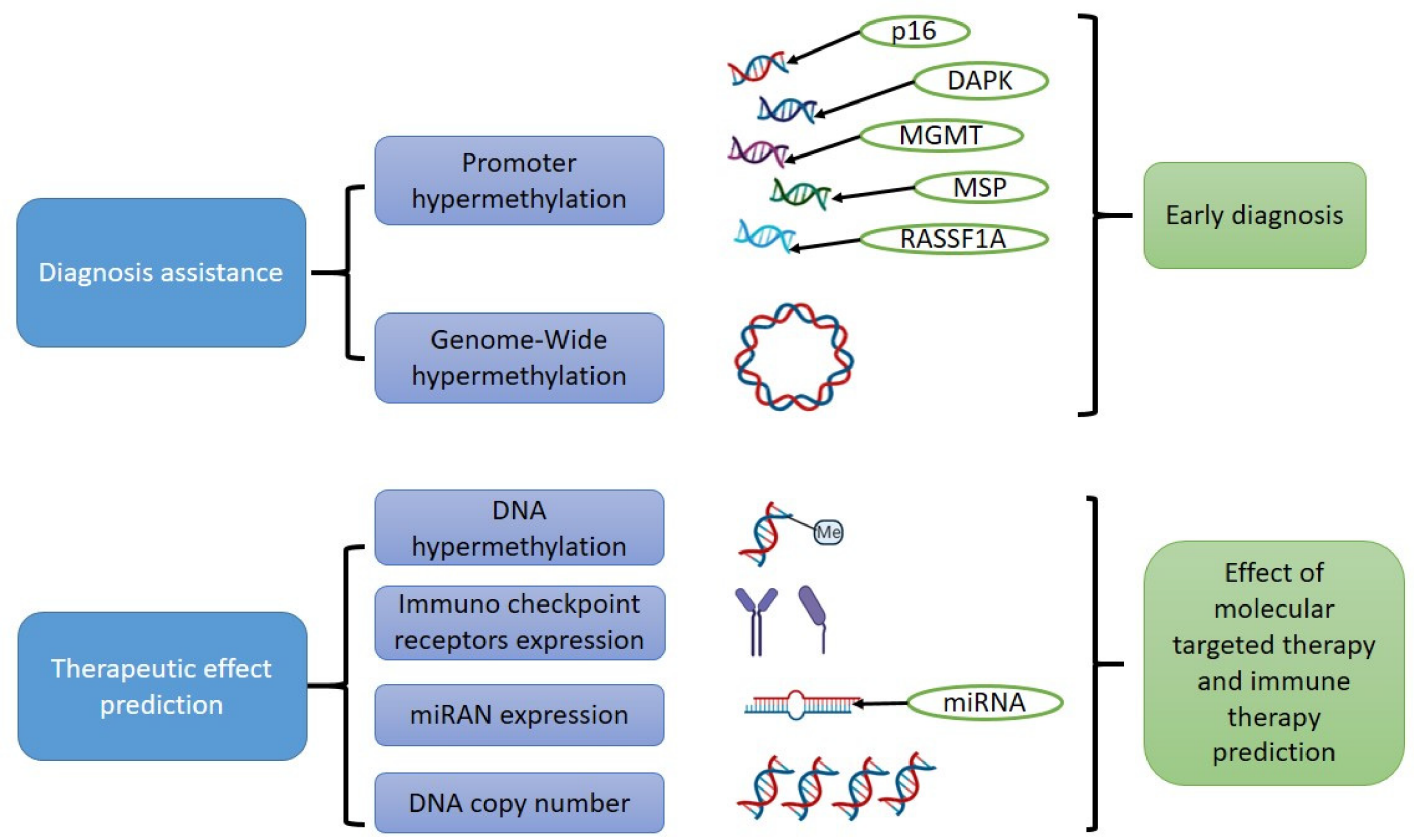
DNA methylation holds promising clinical potential, serving as a novel biomarker for diagnosis and assisting in predicting the therapeutic effects of molecular targeted therapy and immune therapy. Understanding its mechanisms can aid in diagnosing conditions and predicting responses to specific treatments, enhancing personalized medicine approaches.

Hypermethylation of the DAPK, MGMT, MSP tumor suppressor gene (also known as CDKN2A or INK4a), Hypermethylation of the Death‐Associated Protein Kinase (DAPK) gene, methylation of the O‐6‐methylguanine‐DNA methyltransferase (MGMT) gene, hypermethylation of the RAS association domain family 1A (RASSF1A) gene promoter region is well‐documented in various cancers, including HNSCC.^38^, ^39^, ^40^, ^41^ The detection of p16 gene hypermethylation is often used as a biomarker for the early diagnosis and prognosis of HNSCC (Figure 2). Methylation‐specific polymerase chain reaction (MSP) and other molecular techniques are commonly employed to assess the methylation status of the p16 gene in tumor samples.^42^ The presence of p16 hypermethylation may indicate an increased risk of HNSCC, and also it can act as a potential therapeutic target.^38^ Research has shown that the methylation status of the DAPK gene promoter is altered in HNSCC and is associated to its progression, prognosis, and response to therapy.^43^ Detecting the hypermethylation of DAPK can have diagnostic and prognostic implications for HNSCC patients.^44^ MGMT is a DNA repair enzyme that plays a critical role in removing alkyl adducts from the O‐6 position of guanine, which can be induced by DNA‐damaging agents, including chemotherapy.^45^ Methylation of the promoter of the MGMT gene can be silenced, by reducing the repair capacity of the cell and potentially affecting the response treatments inducing DNA damage. In the context of HNSCC, the methylation status of the MGMT gene has been investigated for its impact on treatment outcomes and patient prognosis. Specifically, MGMT promoter methylation has been associated with increased sensitivity to alkylating agents, such as temozolomide, which is used in some cancer treatments.^46^ A tumor suppressor gene called RASSF1A controls cell proliferation, apoptosis, and cell cycle progression. Hypermethylation of the RASSF1A gene promoter region can lead to its inactivation, which may contribute to uncontrolled cell growth and tumorigenesis.^38^ In the context of HNSCC, the methylation status of the RASSF1A gene has been investigated for its potential as a biomarker for disease detection, prognosis, and treatment response. Studies have suggested that RASSF1A hypermethylation may be associated with more aggressive tumor behavior and poorer patient outcomes.^47^

Histone lysine demethylases (KDMs) are enzymes that facilitate the elimination of methyl groups from the lysine residues within histones. By influencing the state of methylation of H3K9, H3K4, H3K27, or H3K36, KDMs contribute to the control of gene transcription, potentially leading to modifications in the levels of expression of tumor suppressor genes and proto‐oncogenes.^48^, ^49^ KDMs exert their influence across various biological processes, encompassing the regulation of the cell cycle, senescence, the response to DNA damage, and the establishment of heterochromatin.^50^ Additionally, they wield significant control over pluripotency. Overexpression of the majority of KDMs has been noted in HNSCC, and impeding their function impacts critical cellular activities such as proliferation, programmed cell death, cellular movement, invasiveness, and the acquisition of stem cell‐like properties. Among the range of KDMs, KDM1, KDM4, KDM5, and KDM6 proteins have emerged as the most promising candidates for prognostic assessment and therapeutic intervention in the context of HNSCC.^51^, ^52^, ^53^, ^54^, ^55^

HNSCC presents a fascinating opportunity to harness benefits of using miRNAs as innovative diagnostic tools. miRNAs, small regulatory nucleic acids consisting of 19–21 nucleotides, have the capacity to interact with and potentially suppress the translation of complementary antisense RNA sequences.^56^ Playing crucial roles in cell proliferation, differentiation, stress response, apoptosis, and immune functions, miRNAs, akin to messenger RNAs, can undergo aberrant expression, contributing to tumorigenesis by modulating the expression of oncogenes or tumor suppressor genes.^57^ These molecules are ubiquitously present in various body fluids and excretions.^58^ After being passively released into bodily fluids, miRNAs can be linked to RNA‐binding proteins, loaded into microvesicles, integrated into exosomes, and discharged into the extracellular space.^59^ Exosomes are 30–120 nm microvesicles that are important for intercellular communication. They make up around 3% of all cell‐free miRNAs. miRNAs in serum and plasma show extraordinary durability because of their carriage by exosomes and binding to RNA‐binding proteins. miRNAs are highly tested because of their tumor‐specific pattern of expression, and stability when in circulation or in body tissues or fluids, and also due to the speed and accuracy of testing in small amounts. Their possible use as cancer biomarkers in the diagnosis, categorization, and prognostic stratification of HNSCC is indicated by the results.^60^

Given the diverse nature of HNSCC and the critical need for early and accurate detection, miRNAs have been extensively studied as potential aids in cancer detection, treatment prognosis, and evaluating the efficacy of treatments. The classification of the function miRNAs' as either oncogenes or suppressors in HNSCC is one important factor worth addressing.^61^ MiR‐21 was first discovered as a possible oncogenic miRNA in HNSCC about a decade ago.^62^ Oncomirs are a subset of miRNAs associated with cancer and are linked to processes like carcinogenesis, malignant transformation, and metastasis. Some oncomirs act as oncogenes, promoting cancerous growth when overexpressed, while others function as tumor suppressors, inhibiting cancer development when their expression is reduced.^63^ These oncomirs play crucial roles in controlling various biological processes including proliferation, migration, and angiogenesis, thereby influencing the initiation and progression of tumors.^64^ It has been determined that a number of particular miRNAs impact the oncogenic pathways in head and neck malignancies. For instance, by suppressing the production of the p53 protein, miRNA‐125a aids in the enhanced proliferation and migration of cancer cells. In contrast, miRNA‐134 affects metastasis and oncogenicity by preventing the WWOX gene from being expressed. It also speeds up cell division by targeting PDCD7 and suppressing the expression of E‐cadherin.^65^, ^66^, ^67^ In tumor samples, tumor suppressor miRNA expression is frequently downregulated. The let‐7 miRNA subfamily is a well‐known family of tumor suppressor miRNAs that regulates normal cell differentiation and development. Head and neck malignancies, among other cancer types, have been associated to reduced let‐7 expression through carcinogenesis.^68^ Let‐7i has been demonstrated to drastically reduce the expression of the chromatin modifier AT‐rich, interacting 3B domain (ARID3B) in the let‐7 group.^69^ Additionally, the oncogene EZH2 upregulates the downregulation of miRNA‐101, which in turn downregulates rap1GAP, another tumor suppressor gene, encouraging head and neck malignancies. The activation of the rap1GAP promoter is suppressed by the trimethylation of H3K27 made possible by EZH2.^70^ Furthermore, head and neck malignancies have been found to have decreased miRNA‐29 levels. Three beta DNA methyltransferases are inhibited by miRNA‐29b, which increases invasiveness by reactivating E‐cadherin expression through promoter area demethylation.^71^ For instance, laryngeal squamous cell carcinoma (LSCC) has been associated to miR‐365a‐3p's role in deregulating the PI3K/AKT pathway.^72^ According to the research, miR‐365a‐3p may act as an oncomir by encouraging growth and metastasis of LSCC through the PI3K/AKT pathway. This provides crucial information on potential pathogenic mechanisms and potential treatment targets for treating LSCC by shedding new light on the intricate processes involving miRNAs and their interactions inside cells. The future of managing HNSCC holds enormous promise for the inclusion of miRNAs as revolutionary diagnostic tools in customized treatment. It has the potential to transform cancer diagnosis, treatment decision‐making, and patient monitoring, resulting in better patient outcomes and a more focused strategy to treat this challenging disease.

Numerous studies have explored the potential of miRNAs as diagnostic indicators, indicating a plausible clinical utility for miRNA signatures specific to HNSCC in bodily fluids. In 2009, Park et al. examined 314 miRNAs in saliva from 50 oral squamous cell carcinoma (OSCC) patients and 50 healthy controls, revealing a down‐regulation of miR‐125a and miR‐200a in oral cancer patients.^73^ Another investigation identified an oral cancer‐specific panel featuring abnormal expression of miR‐375 and miR‐200a, as well as miR‐200c‐141 methylation, capable of distinguishing oral rinse and saliva samples from both OSCC patients and healthy volunteers.^74^

Momen–Heravi and colleagues employed NanoString nCounter technology to assess over 700 miRNAs in saliva samples from OSCC patients, identifying 13 differentially expressed miRNAs in comparison to healthy controls. Among them, 11 miRNAs were down‐regulated (miRNA‐1250, miRNA‐147, miRNA‐136, miRNA‐148a, miRNA‐877, miRNA‐646, miRNA‐668, miRNA‐632, miRNA‐323‐5p, miRNA‐220a, and miRNA‐503), while miRNA‐24, and miRNA‐27b were up‐regulated.^75^ Salazar and colleagues investigated five miRNAs in saliva from healthy controls and HNSCC patients, revealing that miR‐9, miR‐191, and miR‐134 expression could function as novel non‐invasive HNSCC diagnostic biomarkers, validated by data from The Cancer Genome Atlas (TCGA) database.^76^

Examination of miRNAs in plasma samples from tumors and controls demonstrated an up‐regulation of various miRNAs, including miR‐24, miR‐181, miR‐196a, miR‐10b, miR‐187*, in OSCC tissues and plasma compared to control samples.^77^, ^78^, ^79^, ^80^, ^81^, ^82^, ^83^, ^84^, ^85^ Additionally, elevated expression of miR‐155 was observed in tissue and plasma samples of LSCC compared to controls.^86^, ^87^ Conversely, reduced expression of miR‐497 was noted in tissue and plasma of nasopharyngeal carcinoma (NPC) patients relative to noncancerous control patients' plasma.^88^ Furthermore, miR‐17, miR‐20a, miR‐29c, and miR‐223 displayed differential expression in the serum of NPC compared to non‐cancerous controls.^89^

The examination of diverse miRNAs in plasma and serum from HNSCC patients has revealed correlations with prognosis. miR‐130b demonstrated an upregulation in non‐metastatic OSCC samples, confirmed in the plasma of patients without metastasis. Conversely, miR‐296 was identified in metastatic tumors, with its expression validated in the plasma of patients exhibiting metastasis.^90^ A heightened expression of a miRNAs panel, specifically miR‐142‐3p, miR‐374b‐5p, miR‐195‐5p, miR‐186‐5p, and miR‐574‐3p, in the plasma of HNSCC patients associated with a poorer prognosis.^91^, ^92^

Patients with NPC had higher levels of miR‐16, −21, −24, and −155, but lower levels of miR‐378, according to Liu and colleagues' observations. The expression of plasma miRNA was found to be negatively correlated with the advancement of cancer; miR‐21 was statistically significant in both T and N staging, whereas miR‐16 and 24 were significant in N staging alone.^93^, ^94^ In a different study, matched by age, sex, and clinical stage, the serum of NPC patients with shorter survival times and those with longer survival times underwent miRNA profiling. Four serum miRNAs with differential changes were found (miR‐22, miR‐572, miR‐638, and miR‐1234) after identified miRNAs were validated in 512 samples. Patients were categorized into high‐ and low‐risk groups using these miRNAs, with higher risk scores being linked to distant metastasis (DM)‐free survival and worse overall survival (OS) as compared to low‐risk scores.^81^, ^95^

Long non‐coding RNAs (lncRNAs) are a type of RNA molecule that do not code for proteins but have significant regulatory purposes in other cellular processes, such as gene expression, chromatin remodeling, and epigenetic regulation.^96^ Research shows the dysregulation of lncRNAs to be related to development of HNSCC.^97^

HOTAIR, transcribed from the genomic region of the homeobox C gene (HOXC) locus, exhibits a diverse array of functions across various malignancies.^98^ In numerous human cancers, HOTAIR displays abnormal expression patterns, rendering it a potential biomarker with prognostic value.^99^ Operating as an oncogene, HOTAIR recruits EZH2 to catalyze the trimethylation of histone H3 at lysine 27 (H3K27me3), thereby repressing downstream tumor suppressor genes.^100^ Augmentation of HOTAIR transcription is facilitated by STAT3 through its interaction with pEZH2‐serine21, fostering heightened cellular growth in HNSCC by activating the PI3K/AKT pathway.^101^ Perturbation of HOTAIR and EZH2 also triggers apoptosis linked to mitochondria and curtails HNSCC growth. Furthermore, elevated HOTAIR levels have been associated with unfavorable prognoses in patients with LSCC, attributed to its role in inducing PTEN methylation. Additionally, HOTAIR exerts an upregulatory effect on stanniocalcin‐2 (STC2) by sequestering miR‐206 and activating the PI3K/AKT signaling cascade. This concerted action promotes enhanced proliferation, invasion, and migration of HNSCC cells.^99^, ^102^, ^103^

Metastasis‐associated lung adenocarcinoma transcript 1 (MALAT1) is an abundant lncRNA primarily located in the cell nucleus, with prevalent upregulation observed in both primary tumors and metastatic tissues of patients. Research employing chromatin immunoprecipitation (ChIP) and luciferase reporter assays has revealed the binding of STAT3 to the promoter region of MALAT1, subsequently leading to the transcriptional activation of MALAT1.^104^ This activated MALAT1 then establishes a reciprocal interaction with miR‐30a, propelling the process of epithelial‐mesenchymal transition (EMT) and hastening the metastatic progression of HNSCC. MALAT1 operates as a regulator of cellular processes by targeting p21 (RAC1)‐activated kinase 1 (PAK1) through the suppression of miR‐140‐5p.^105^ This regulatory mechanism further fuels the proliferation, migration, and invasion of tongue squamous cell carcinoma (TSCC) cells both in controlled laboratory settings and in live animal models. Importantly, MALAT1 exhibits a similar promotive effect on the migration and invasion of Hep‐2 cells in LSCC and FaDu cells in hypopharyngeal squamous cell carcinoma (HSCC).^106^

HOTTIP (HOXA transcript at the distal tip) is a lncRNA that plays a role in gene expression regulation by interacting with chromatin‐modifying complexes and facilitating the activation of specific target genes, particularly those within the HOXA gene cluster. Dysregulation of HOTTIP has been associated with tumor progression in HNSCC, likely by influencing the expression of HOXA genes and potentially impacting cell differentiation and development.^107^

AFAP1‐AS1 (Actin Filament Associated Protein 1 Antisense RNA 1) is a lncRNA that is antisense to the gene encoding Actin Filament Associated Protein 1 (AFAP1). It is involved in gene expression regulation and has been shown to interact with various cellular components.^108^ AFAP1‐AS1 has been implicated in HNSCC, where it is often upregulated. In OSCC, AFAP1‐AS1 upregulation has been associated with tumor progression, invasion, and poor prognosis. It may contribute to cancer cell proliferation, migration, and metastasis.^109^

Cancer susceptibility 9 (CASC9) a lncRNA called CASC9 has been connected to the emergence and spread of cancer. Through interactions with proteins and other regulatory molecules, it affects the control of genes and cellular processes.^110^ CASC9 has been implicated in HNSCC, where it is often upregulated and hence, CASC9 overexpression might aid in the migration, multiplication, and spread of cancer cells.^111^

According to the Guan GF et al., H19 exhibits a substantial upregulation in a set of 65 primary tumor samples and two cell lines specific to head and HNSCC, assuming a pivotal role in the facilitation of tumor growth and advancement.^112^ Notably, H19 expression emerges as more pronounced in tumors that have undergone metastasis compared to those that remain non‐metastatic. This pattern is consistently mirrored in TSCC cells, which display higher H19 levels in contrast to regular human squamous cells. Functioning as a competitive endogenous RNA (ceRNA), H19 operates as a molecular sponge for miRNA let‐7a, effectively sequestering it. This sequestration results in the elevation of let‐7a's target, HMGA2, a significant orchestrator of tumor metastasis.^113^ In the context of LSCC, H19's upregulation is linked to the targeting of miR‐148a‐3p, fostering an augmentation in the expression of DNA methyltransferase 1. Consequently, the heightened presence of H19 contributes to an elevation in cellular DNA methylation levels.^114^

GAS5 is a lncRNA that was initially identified as being upregulated during growth arrest. It has been demonstrated to play a role in the control of a number of cellular functions, including the progression of the cell cycle, apoptosis, and stress response.^115^ Dysregulation of GAS5 has been implicated in HNSCC. GAS5 downregulation has been associated with altered cell cycle regulation and apoptosis, potentially contributing to tumor growth and survival.^116^

However, the clinical use of RNA quantification levels as biomarkers faces challenges due to inconsistent reports on its utility. Lack of reproducibility may result from lacking multi‐center studies and adequately powered cohorts. Additionally, variations in techniques, such as sample types and sources, sample collection timing, purification methods, and RNA extraction methods, contribute to the lack of consensus. Blood collection and processing present sensitive steps with potential RNA contamination, influencing downstream technologies. Accurate RNA quantification is hindered by low RNA quantities and interference from contaminating salts and proteins in biological fluids. Standardizing experimental conditions and validating findings in independent cohorts and verification studies are crucial steps before establishing the clinical utility of circulating RNAs, particularly in disease monitoring and treatment decisions. Addressing these issues is essential to prevent biased results in circulating RNA studies.^117^

Exploring mRNAs as markers in the bodily fluids of HNSCC patients for diagnosis and prognosis has been limited due to their known low stability and susceptibility to RNase degradation, posing challenges for their effectiveness as liquid‐biopsy marker. However, Li et al. addressed this issue by demonstrating the possibility to assess mRNA in saliva samples. A comprehensive analysis of the transcriptome in 32 salivary samples of OSCC patients and 32 normal matched individuals, identified seven upregulated mRNA biomarkers, viz. IL8, SAT, DUSP1, IL1b, HA3, S100P, and OAZ1, which are related to cancer. Validation studies confirmed the consistent and reliable detection of the potential salivary RNA biomarkers. The four salivary mRNAs, (IL1b, IL8, SAT, and OAZ) together exhibited 91% specificity and sensitivity for detecting oral cancer, underscoring the usefulness of salivary transcriptome diagnostics in detection of oral cancer.^118^

Building on these discoveries, subsequent studies sought to confirm the results across different uses and cohorts. One study assessed the discriminatory capability of the seven mRNAs (IL8, SAT, DUSP1, IL1b, HA3, S100P, and OAZ1) in distinguishing patients with OSCC from that of the healthy individuals in five independent case–control cohorts involving 395 subjects. The seven mRNAs in OSCC had increased expression compared to the controls across all cohorts, with statistically significant elevation in IL‐8 and SAT, validated the earlier findings from Li and colleagues. Another recent study investigated the presence of IL1b, IL8, SAT, and OAZ in the saliva of 34 patients with primary OSCC (T1N0M0/T2N0M0), 20 patients with oral leukoplakia and dysplasia, and 31 matched healthy‐control subjects for prior detection of OSCC. The results demonstrated a prediction likelihood of 80% for patients with OSCC, and the SAT and IL‐8 biomarkers exhibited a high predictive ability of 75.5%, proposing the potential use of these two biomarkers in a cost‐effective model of prediction for OSCC patients.^119^

Additional studies have supported the viability of evaluating mRNA in bodily fluids, although they are relatively limited.^120^, ^121^ Aggarwal and colleagues observed significantly elevated levels of gal‐1 and gal‐3 mRNA in serum and tumor sites of OSCC patients relative to controls. Furthermore, both galectins were much more expressed in patients with larger tumor loads, allowing for >80% sensitivity and specificity in the classification of patients from healthy subjects.^120^ In a different investigation, the transgelin mRNA levels in the tissue, serum, and saliva of patients with OSCC and negative controls were examined. Serum transgelin mRNA expression did not differ significantly between OSCC patients and healthy controls; however, OSCC tissue and saliva contained higher concentrations of this marker. These elevated levels were associated with clinicopathological parameters, indicating their potential as promising biomarkers for OSCC and independent prognostic factors.^121^ It has recently been established that the HPV poses a serious risk for HNSCC cancers, especially those that arise in the oropharynx. Oral rinses containing saliva from 82 HNSCC patients with tumor p16(INK4a) status were tested for the presence of HPV‐16 RNA. Of the 40 patients with p16(INK4a)‐positive tumors, 24 had HPV‐16 mRNA discovered in their oral rinse samples; patients with p16(INK4a)‐negative tumors did not have any HPV‐16 mRNA detected in their samples.^122^ Despite being transcribed from genes, non‐coding RNAs—such as miRNAs and lncRNAs—do not encode proteins; instead, they control the expression of coding genes. These molecules exhibit sustained circulation in human bodily fluids and can be accessed through non‐invasive means, making them attractive candidates for biomarkers.^123^

In Table 1, we list a few epigenetic biomarkers, the roles and mechanisms, and the implication at work in HNSCC. Due to their interactions with particular genes, the epigenetic biomarker described herein should be categorized as either oncogenes or suppressors. Recognizing the more complex issue of epigenetic impacts, which affect entire signaling cascades rather than just one gene, is crucial.

**TABLE 1:** Epigenetic biomarkers roles, mechanism, and implications for HNSCC.

Epigenetic biomarker	Roles and mechanisms	Implications for HNSCC	References
p16 Gene Hypermethylation (CDKN2A/INK4a)	Tumor suppressor gene; hypermethylation leads to gene inactivation; early diagnosis, prognosis, potential therapeutic target	Biomarker for early diagnosis and prognosis; potential therapeutic target	42
DAPK Gene Hypermethylation	Associated with tumor progression, prognosis, response to therapy	Diagnostic and prognostic implications; guides treatment strategies	43
MGMT Gene Methylation	DNA repair enzyme; methylation silences gene; affects repair capacity; impacts response to DNA‐damaging treatments	Predicts treatment outcomes and patient prognosis; sensitivity to alkylating agents like temozolomide	46
RASSF1A Gene Hypermethylation	Tumor suppressor gene; hypermethylation leads to inactivation; associated with uncontrolled cell growth	Biomarker for disease detection, prognosis, and treatment response; linked to more aggressive tumor behavior	38, 41, 47
KDM1	Impacts gene transcription and cellular processes	Promising for prognostic assessment and therapeutic intervention	51
KDM4	Influences gene transcription and cellular functions	Potential role in prognostic assessment and therapeutic intervention	52
KDM5	Modulates gene expression and biological processes	Considered for prognostic assessment and therapeutic intervention	53
KDM6	Affects gene transcription and cellular properties	Promising for prognostic assessment and therapeutic intervention	51, 55
miR‐21	Oncogenic miRNA; promotes cancerous growth when overexpressed	Potential diagnostic marker; associated with carcinogenesis, malignant transformation, and metastasis	62
miRNA‐125a	Suppresses p53 protein production; enhances proliferation and migration of cancer cells	Contributes to enhanced proliferation and migration of cancer cells	65, 66
miRNA‐134	Suppresses WWOX gene expression; affects oncogenicity and metastasis	Implicated in oncogenicity, metastasis, and cell division regulation	67
let‐7 family	Tumor suppressor miRNAs; regulate normal cell differentiation and development	Reduced expression associated with carcinogenesis; potential diagnostic and therapeutic implications	68, 69
miRNA‐101	Downregulates rap1GAP, a tumor suppressor gene; affected by oncogene EZH2	Impacts head and neck malignancies through suppression of tumor suppressor gene expression	70
miRNA‐29b	Decreases levels of three beta DNA methyltransferases; reactivates E‐cadherin expression	Inhibition of DNA methyltransferases may contribute to invasiveness through promoter area demethylation	71
HOTAIR	Oncogenic lncRNA; recruits EZH2 to repress tumor suppressor genes	Potential diagnostic biomarker	99
	Facilitates histone H3 trimethylation (H3K27me3) through EZH2	Prognostic value for patient outcomes	100
	Enhanced transcription by STAT3 interaction with pEZH2‐serine21	Target for therapeutic intervention to inhibit oncogenic pathways	101
	Activation of PI3K/AKT pathway; cellular growth in HNSCC	Contributes to tumor growth and progression	101
	Upregulation of stanniocalcin‐2 (STC2); activation of PI3K/AKT signaling cascade	Promotes proliferation, invasion, and migration of HNSCC cells	102, 103
MALAT1	Abundant lncRNA; prevalent upregulation in primary tumors and metastatic tissues	Potential diagnostic and prognostic marker	104
	STAT3 binding to promoter region; transcriptional activation	Influence on metastatic progression of HNSCC	104
	Reciprocal interaction with miR‐30a; promotes epithelial‐mesenchymal transition (EMT	Role in epithelial‐mesenchymal transition and metastasis	105
	Targets p21 (RAC1)‐activated kinase 1 (PAK1) through suppression of miR‐140‐5p	Facilitates proliferation, migration, and invasion of TSCC cells	105
HOTTIP	Facilitates activation of specific target genes; associated with tumor progression	Impacts HOXA gene expression and cell differentiation	107
	Regulation of gene expression through chromatin‐modifying complexes	Implication in tumor progression and cell development	107
AFAP1‐AS1	Antisense to AFAP1 gene; involved in gene expression regulation	Upregulation associated with tumor progression, invasion, and poor prognosis	108
	Interaction with various cellular components	Potential role in cancer cell proliferation, migration, and metastasis	108, 109
CASC9	Implicated in cancer development; interacts with proteins and regulatory molecules	Upregulation linked to cancer emergence, migration, multiplication, and spread	110, 111
H19	Upregulation associated with tumor growth and advancement; role as a ceRNA	Potential diagnostic marker	113
	Promotes tumor growth and metastasis through HMGA2 upregulation	Prognostic implications for metastatic tumors	113
	Targets miR‐148a‐3p; contributes to DNA methyltransferase 1 expression	Induces cellular DNA methylation	114
GAS5	Regulates cell cycle progression, apoptosis, and stress response	Dysregulation linked to altered cell cycle regulation and apoptosis	115
	Upregulated during growth arrest	Potential role in tumor growth and survival	116

## 4 TREATMENTS IN PERSONALIZED MEDICINE

### 4.1 Targeted therapies

A form of cancer treatment known as “targeted therapy” concentrates on particular molecules or pathways which are important for the growth and spread of cancer cells. The development of targeted therapies is often based on a deeper understanding of the molecular and genetic changes that drive cancer growth. Scientists identify specific proteins, receptors, or genetic mutations that are overexpressed or abnormally active in certain types of cancer. These unique characteristics become the targets for these therapies. There are several types of targeted therapies used in cancer treatment.

#### 4.1.1 Immunotherapy

In personalized HNSCC treatment, immune responses can be influenced by targeting specific immune‐related intracellular pathways with immunotherapy. Researchers have been studying immune checkpoint inhibitors (ICIs) that target co‐stimulatory and co‐suppressive checkpoints on immune cells as potential treatment targets.^124^ One promising approach involves using the anti‐LAG‐3 antibody, BMS‐986016, in combination with nivolumab for metastatic solid tumors, including HNSCC.^125^, ^126^ Additionally, eftilagimod alpha (efti), a soluble LAG‐3 protein, has been investigated to stimulate dendritic cells and enhance T cell recruitment, potentially leading to stronger anti‐tumor responses in combination with pembrolizumab.^127^ TACTI‐002, has shown results with efti plus pembrolizumab in a phase II treatment study for PD‐L1 unselected metastatic HNSCC patients.^128^ Another strategy involves targeting IDO‐1, an enzyme which induces immunotolerance. While a phase I/II trial combining an IDO‐1 inhibitor, epacadostat, with pembrolizumab in HNSCC showed enhanced response rates.^129^ Further development of this approach was halted after negative results in melanoma Phase III studies.^130^ Targeting the EGFR pathway has been an established therapeutic strategy in HNSCC. Combining pembrolizumab with cetuximab, an EGFR‐targeted antibody, demonstrated promising activity in platinum‐refractory or ‐ineligible R/M HNSCC patients.^131^ Additionally, the anti‐EGFR TKI, afatinib, has been shown to synergize with immunotherapy in HNSCC patients.^132^ Modulating angiogenesis in the tumor microenvironment has also been explored as a potential synergistic approach with immunotherapy. Lenvatinib, a multikinase inhibitor targeting VEGFR, FGFR, PDGFR‐α, RET, and KIT, in combination with pembrolizumab, showed improved response rates and manageable toxicity in a Phase Ib/II trial.^133^ Epigenetic modification is another area of interest, with the HDAC inhibitor vorinostat combined with pembrolizumab showing promising results in R/M HNSCC and salivary gland cancer patients.^134^ The inducible T cells co‐stimulator (ICOS) pathway, including the ICOS agonist GSK3359609, have also been investigated in HNSCC. Combining GSK3359609 with pembrolizumab demonstrated promising anti‐tumor activity in patients with anti‐PD‐1/PD‐L1‐naïve HNSCC.^135^ Furthermore, other co‐stimulator/co‐inhibitor targets, such as TIM‐3 and KIR, combined with anti‐PD‐1 therapy, are also being explored in various advanced solid tumors.^136^ Personalized treatment approaches for HNSCC are focusing on targeting specific immune‐related pathways and combining various immunotherapies to increase anti‐tumor responses and provide a better patient outcome.

Current evidence has shown that many different approaches for HNSCC immunotherapy have been utilized in PM; these strategies include oncolytic virus therapy, costimulatory agonists, ICIs, adoptive T cell transfer (ACT), antigenic vaccines, and EGFR‐targeted therapy. The proper application of these immunotherapy techniques in the management of HNSCC provides major benefits for patients, as each of these approaches has particular advantages of its own.^137^

The underlying principle of antitumor immunotherapy is that immunological escape is made possible by changes in the tumor microenvironment and immune surveillance. Antitumor immunotherapy in HNSCC is justified based on a number of important findings. Firstly, HNSCC exhibits a relatively high tumor mutation burden (TMB).^138^ This is significant since the effectiveness of ICIs has been connected to a high TMB. The posited method is the synthesis of modified proteins possessing antigenic characteristics from mutant DNA, acting as targets for immune cells within the tumor.^139^ In malignancies caused by the HPV, mutagenesis is linked to the activity of the apolipoprotein B mRNA editing catalytic polypeptide‐like (APOBEC) proteins during gene editing. Particularly, APOBEC3B, APOBEC3D, APOBEC3C, APOBEC3G, APOBEC3H, and APOBEC3F are the viral response genes that are more expressed in HPV‐related HNSCC than in HPV‐negative HNSCC.^140^, ^141^ The COSMIC database has classified the C → T and C → G mutations as signatures 2 and 13, respectively, resulting in a clustered pattern of APOBEC enzymatic activity. Higher hydrophobicity in neopeptides translated from APOBEC‐mutated sequences indicates heightened immunogenicity and is correlated with an ICI response.^142^ Conversely, enhanced ICI responsiveness is also associated with tobacco mutagenesis and methylation patterns.

Secondly, although inflammation may have a role in the emergence of HNSCC,^142^ HNSCC can exhibit immunosuppressive characteristics. Due to poor antigen‐presenting ability, upregulation of PD‐1 and other ICIs, and decreased natural killer cells, many HNSCC patients have defective tumor‐infiltrating T lymphocytes.^143^ Thirdly, immune cells that may be targeted for anti‐tumor actions frequently infiltrate HNSCC. Fourth, HPV is responsible for a growing fraction of HNSCC cases, indicating a breakdown in the immune system's ability to regulate this persistent viral infection and offering a useful therapeutic and antigenic target. Targetable, the PD‐1/PD‐L1 pathway is a crucial immune escape mechanism employed by malignancies. Anti‐PD1/PD‐L1 drugs boost the anti‐tumor immune response by interfering with the immunosuppressive signals of malignancies.^144^, ^145^ Recent large‐scale clinical trials have provided biological justification for targeting the PD‐1/PD‐L1 pathway in HNSCC, as seen by improved outcomes with ICIs as compared to standard‐of‐care therapy.

In 361 patients with recurrent HNSCC who had progressed within 6 months following platinum‐based chemotherapy, Ferris et al. carried out a Phase III open‐label, randomized trial to assess the efficacy of nivolumab versus conventional single‐agent systemic therapy. OS was the main result, and was followed by progression‐free survival (PFS), safety, objective response rate, and quality of life as indicated by the patient. Patients were randomly assigned to receive nivolumab (3 mg per kg of body weight) every 2 weeks or standard single‐agent systemic therapy. The median OS in the nivolumab group was 7.5 months (95% CI: 5.5–9.1), compared to 5.1 months in the standard treatment group. Nivolumab had a greater 1‐year survival rate than conventional therapy (36.0% vs. 16.6%). The median PFS with nivolumab was 2.0 months (95% CI: 1.9–2.1) compared to 2.3 months with standard treatment. The 6‐month PFS rate with nivolumab was 19.7% versus 9.9% with conventional treatment. Nivolumab had 13.3% response compared to 5.8% for conventional therapy. Treatment‐related Grade 3 or 4 adverse events occurred in 13.1% of nivolumab patients versus 35.1% in conventional treatment. Nivolumab maintained physical, role, and social functioning, while conventional therapy worsened it. Thus, the study showed that OS of platinum‐refractory, recurrent HSNCC patients treated with nivolumab was longer than with single‐agent therapy.^146^ Patil et al.^147^ conducted a randomized Phase III superiority study to evaluate whether the incorporation of low‐dose nivolumab into triple metronomic chemotherapy (TMC) resulted in enhanced OS. Patients were divided equally into two groups: one receiving TMC comprising oral methotrexate 9 mg/m^2^ weekly, celecoxib 200 mg twice daily, and erlotinib 150 mg once daily; and the other receiving TMC combined with intravenous nivolumab (TMC‐I) at a flat dose of 20 mg administered once every 3 weeks. The primary outcome measure was the OS rate at 1 year. This study showed that, the addition of low‐dose nivolumab to metronomic chemotherapy demonstrated enhanced OS, presenting itself as a viable alternative standard of care for individuals unable to obtain full‐dose checkpoint inhibitors.^147^

Cohen et al. conducted a Phase III, randomized, open‐label trial at 97 medical centers in 20 countries to compare the effectiveness of pembrolizumab and standard‐of‐care therapy for HNSCC. Intravenous pembrolizumab 200 mg every 3 weeks or the investigator's choice of standard doses of docetaxel, cetuximab, or methotrexate or were given to patients with HNSCC who progressed during or after platinum‐containing treatment for recurrent or metastatic disease, or within 3–6 months of platinum‐containing multimodal therapy for locally advanced disease. A total of 247 patients were randomly assigned to pembrolizumab and 248 to standard treatment. On May 15, 2017, 181 (73%) of 247 pembrolizumab patients and 207 (83%) of 248 standard‐of‐care patients had died. With pembrolizumab, the median OS was 8.4 months (95% CI: 6.4–9.4) and with standard of care, 6.9 months (5.9–8.0) (hazard ratio 0.80, 0.65–0.98; nominal *p* = .0161). Pembrolizumab caused fewer Grade 3 or greater adverse events than standard of care, specifically 33 out of 246 (13%) versus 85 out of 234 (36%). The most common treatment‐related side events were hypothyroidism with pembrolizumab (33 [13%]) and fatigue with usual care (43 [18%]). Four pembrolizumab patients (due to unspecified reasons, Stevens–Johnson syndrome, perforations in large intestine, and malignant neoplasm progression) and two of standard care patients (pneumonia and malignant neoplasm progression) died during treatment. Pembrolizumab's clinically significant extension of OS and favorable safety profile in recurrent or metastatic HNSCC patients warrant its use as monotherapy and in combination therapy in earlier stages.^148^ In addition, advanced cutaneous squamous cell carcinoma and recurrent or metastatic HNSCC can be treated with PD‐1‐targeted cemiplimab. FDA‐approved durvalumab targets the PD‐L1 receptor for unresectable stage III NSCLC and locally progressed stage III HNSCC. Ipilimumab, a CTLA‐4 receptor inhibitor, is FDA‐approved for unresectable or metastatic melanoma.^137^ By targeting immune system receptors, these monoclonal antibodies demonstrate cancer treatment advances by using the body's immunological response to fight diverse cancers.

#### 4.1.2 Gene silencing and editing

Exploring gene silencing and editing techniques represents potential strategies in comprehending HNSCC development, identifying therapeutic targets, and developing novel treatments. This involves the suppression of specific genes critical to neoplasm growth. CRISPR/Cas9 and RNA interference (RNAi) or post‐transcriptional gene silencing (PTGS) are novel approaches in targeting these conditions. CRISPR/Cas9 is a revolutionary gene‐editing technology, which aims to specifically target genetic alterations that drive tumor growth. While RNAi or PTGS can provide a temporary knockdown of targeted mRNA, achieving a permanent knockout of the relevant genes is possible via gene editing techniques.

##### RNA interference

Studies have demonstrated that sequence‐specific RNA templates can be created to effectively downregulate the expression of E6 and/or E7 oncoproteins while upregulating tumor‐suppressor proteins like p53 and pRb in the context of HNSCC associated with high‐risk human papillomavirus (HR‐HPV).^149^, ^150^, ^151^ Studies on RNAi against HPV have traditionally been carried out on animal models or cervical cancer cells. However, employing E6‐ and/or E7‐targeting short hairpin RNAs (shRNA) or small interfering RNAs (siRNAs), positive outcomes have been seen in HR‐HPV HNSCC cell lines, both in vitro and in vivo.^151^ Oncogenes E6 and/or E7 have been significantly inhibited by these strategies. Research has shown that a link exists between HR‐HPV and HNSCC. It is necessary to enhance the delivery of RNA‐based therapeutics to the tumor site if individualized HNSCC treatments are to completely realize the therapeutic potential of RNAi. In order to effectively distribute anti‐E6/E7 siRNAs, lipid nanoparticles coated with anti‐EGFR antibodies have been studied.^152^ This innovative method was shown to significantly inhibit viral oncogenes and induce apoptosis, which results in potent anticancer activity both in vitro and in vivo. By tailoring the RNAi method to target specific genetic alterations present in individual patients' head and neck tumors, personalized treatments can be developed. The use of sequence‐specific siRNAs or shRNAs against unique molecular targets in each patient's cancer cells could enhance treatment effectiveness and reduce off‐target effects. Harnessing the potential of RNAi to target HR‐HPV‐associated oncoproteins in personalized HNSCC treatments shows promising results. Advancements in delivery technologies, such as the use of lipid nanoparticles coated with targeting antibodies, are bringing us closer to the realization of more effective and precise therapies for patients with this devastating disease.^151^

##### CRISPR/Cas9

The CRISPR/Cas9 system stands out for its robustness and versatility. To unleash the full potential of CRISPR/Cas9 for personalized HNSCC treatments, it is essential to develop safe and efficient delivery vehicles. Liposomes or lipid nanoparticles have emerged as strong candidates for this purpose due to their ability to prolong circulation time, improve targeted delivery, enhance cellular uptake, reduce immunogenicity, and minimize toxicity.^153^ In the context of HR‐HPV‐related HNSCC, targeting HPV E6/E7 genes can be a logical approach to suppress tumor growth. The use of CRISPR/Cas9 with specific guide RNAs (sgRNAs) can lead to the inactivation of the E6/E7 genes, inducing the reactivation of tumor suppressors like p53 or Rb, resulting in cell cycle arrest and cell death.^154^, ^155^, ^156^ This approach has shown promise in inhibiting tumor growth in preclinical studies. Efficient and tumor‐specific delivery of CRISPR/Cas9 to the HNSCC cells is key for successful personalized treatments. Lipid nanoparticles have been explored as delivery vectors for CRISPR/Cas9 targeting HPV E6/E7 in cervical cancer, resulting in tumor elimination and improved survival rates.^157^, ^158^ Abboodi et al.'s recent investigation shed light on the behavior of HPV‐16‐transformed cells, where they discovered that the levels of E7 mRNA drastically dropped when they employed CRISPR/Cas9 to eliminate the p53 gene in these cells. This suggests that HPV‐16‐transformed cells might have the ability to thrive without continuous expression of viral oncogenes if their p53 gene is disrupted. However, it remains unclear whether knocking down or knocking out HPV E6/E7 in HPV‐dependent cells with intact p53 can lead to the emergence of HPV‐inactive cells that can still proliferate.^104^ This potential outcome needs to be thoroughly monitored and studied to understand its implications. Other nanoparticle‐based systems, Poly (β‐amino ester)‐based polyplex nanoparticles and pH‐responsive cationic liposomes, have also demonstrated efficacy in delivering CRISPR/Cas9 to target HPV genes.^158^, ^159^ To optimize the CRISPR/Cas9 system further, researchers have explored modifications to the Cas9 enzyme or tested related enzymes like Cas13 to improve editing efficiency and specificity.^160^, ^161^ Such advancements may help overcome challenges related to off‐target effects and increase the precision of HPV oncogene knockout. Personalized HNSCC treatments leveraging the CRISPR/Cas9 system offer exciting possibilities. Effective delivery systems, such as lipid nanoparticles, can be tailored to target specific tumors, enabling the precise and permanent knockout of HPV oncogenes.^157^ With ongoing research and refinement of gene editing technologies, personalized CRISPR/Cas9‐based therapies hold the potential to revolutionize the treatment landscape for HR‐HPV‐related HNSCCs.^162^ However, further investigations and clinical trials are needed to fully assess the efficacy and safety of these approaches in the context of personalized cancer treatments.

Personalized HNSCC treatments can influence the CRISPR/Cas9 system for precise and safe gene manipulation to upregulate p53 expression and restore p53 bioavailability. This approach holds potential as a therapy for HR‐HPV‐driven cancers, where the HPV oncogene E6 promotes the degradation of p53, contributing to the carcinogenic process.^163^ Instead of using adenoviral vectors to deliver wild type p53, which was the focus of early attempts, researchers can now utilize CRISPRa/dCas9, a variant of the CRISPR system, for activation of p53.^164^ This system allows for targeted activation of the p53 gene, enhancing its expression levels in cancer cells.

Patients with HNSCCs caused by HR‐HPV would undergo thorough profiling to identify specific genetic alterations and the status of their p53 gene. Based on the patient's genetic profile, a specific guide RNA (gRNA) would be designed to target the regulatory region of the p53 gene.^165^ The CRISPRa/dCas9 system would be engineered to carry the gRNA and the necessary activation domains. Before treatment, cancer cells from the patient would be collected and tested in vitro to verify the efficacy and safety of the CRISPRa/dCas9 system in activating the p53 gene.^165^ This step would also help ensure the specificity of the treatment. Once the in vitro testing is successful, the personalized CRISPRa/dCas9 system would be introduced into the patient's cancer cells in vivo.^163^ This could be achieved through direct injection into the tumor site or using delivery methods such as nanoparticles or viral vectors. The patient's response to the treatment would be closely monitored, with regular assessments of tumor size, recurrence rates, and OS. Long‐term follow‐up would be conducted to determine the treatment's effectiveness over time.

In the case of HPV+ HNSCC, a promising approach is to silence or edit the viral oncogenes HPV E6 and E7, which are known to be significant contributors to tumor development. Techniques like RNAi or CRISPR/Cas9 can be used to accomplish this. The potential issue with this strategy is that when HPV viral gene expression is inhibited, it is possible for HPV‐negative HNSCC to arise. Contrary to popular belief, certain HPV DNA‐positive HNSCC cases do not express the HPV E6/E7 oncogenes, and these tumors have worse OS and greater recurrence rates than HPV‐active HNSCC tumors. The exact cause of this variation is unknown.^166^, ^167^

### 4.2 Stem cell therapies

**FIGURE 3:**
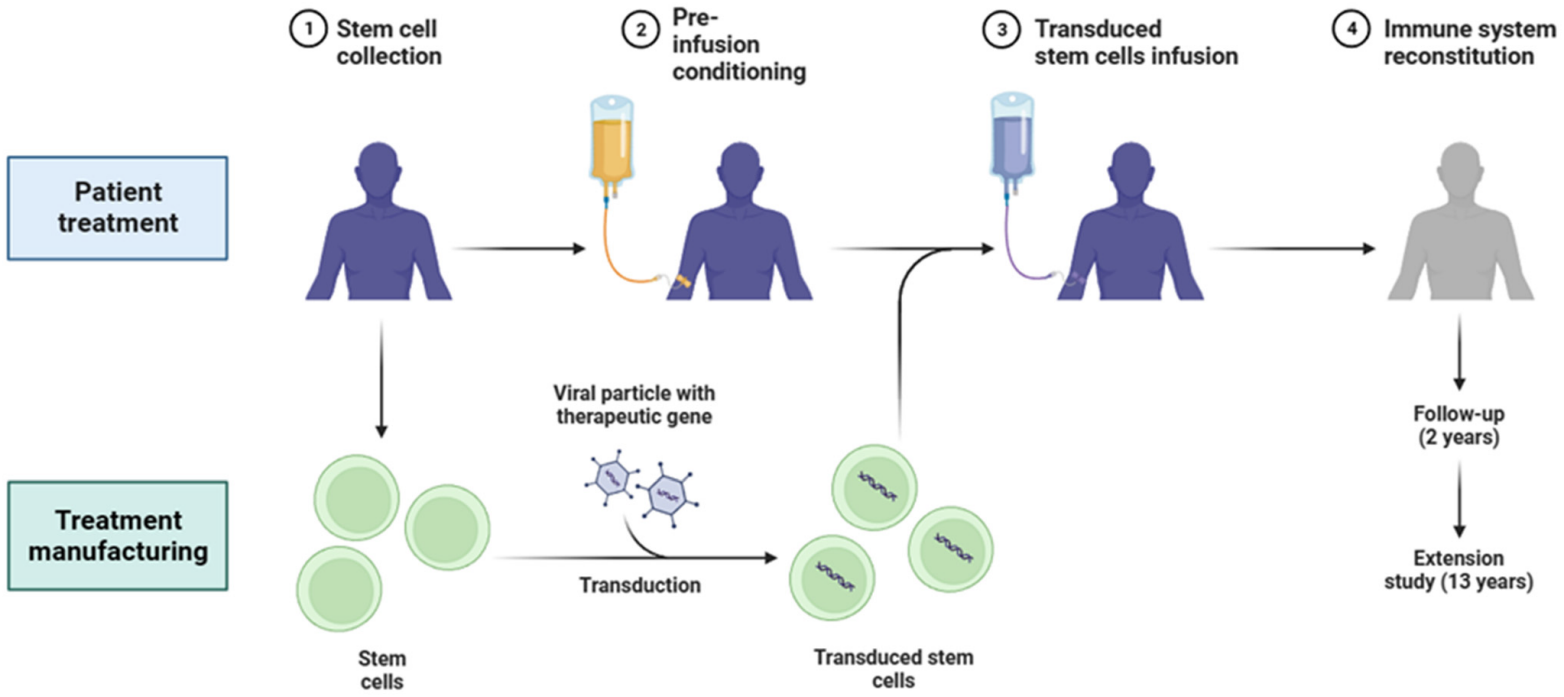
Basic treatment phases involved in gene therapy.

Personalized HNSCC treatments can be developed based on the identification and understanding of specific markers and downstream pathways associated with cancer stem cells (CSCs) and other therapeutic targets. Here's how this information can be utilized to design personalized treatment approaches (Figure 3).

The presence of specific markers like CD44, Bmi‐1, FoxM1, and ALDH in CSCs provides potential targets for therapy.^168^ Researchers can work on creating drugs or therapies that target these markers, aiming to inhibit CSC activity, diminish self‐renewal, and potentially sensitize CSCs to alternative treatments or novel therapeutic approaches. Inhibiting pathways downstream of CSC markers, such as the focal adhesion kinase (FAK) pathway associated with integrin β1, or the FGF signaling pathway, can be explored as a strategy to target CSCs or enhance the sensitivity of CSCs to conventional treatments.^169^ Antibody‐drug conjugates, like MEDI0641, which specifically target oncofetal antigens like 5T4, have shown promising results in reducing CSC populations and limiting tumor recurrence.^170^ Similar targeted antibody‐based therapies can be explored to attack CSCs in HNSCCs. Inhibition of histone deacetylases (HDACs) has been shown to disrupt CSCs and sensitize tumor cells to cisplatin treatment. Understanding the epigenetic alterations that regulate CSCs can lead to the development of epigenetic therapies personalized for individual patients.^171^ Targeting transcription factors like brachyury or SOX2, which play critical roles in regulating CSCs in certain HNSCCs, can be investigated to develop targeted therapies that suppress CSC properties and enhance sensitivity to chemotherapy or radiotherapy.^172^ Given the heterogeneity of HNSCCs, a personalized treatment approach may involve combinations of therapies targeting different pathways and markers. The study by Leong et al. demonstrated that inhibiting these receptors reduced the levels of ALDH+ cells, which are associated with CSCs. Targeted therapies against EGFR and IGF‐1R could be considered for patients whose tumors exhibit overexpression or activation of these receptors. Identifying the presence of these receptors in a patient's tumor through molecular testing can help determine the potential effectiveness of such therapies.^173^ Isoorientin was shown to inhibit stemness in oral cavity cell lines through the STAT3/Wnt/β‐catenin axis. This finding suggests that patients with tumors exhibiting high STAT3 or Wnt/β‐catenin activity may benefit from treatments involving isoorientin or other agents that target these pathways. Molecular profiling of tumors can help identify patients who could respond well to this type of therapy.^174^ COX‐2 promotes the maintenance of CSCs, and inhibiting this enzyme resulted in decreased expression of CSC‐associated genes and reduced sphere formation in hypopharyngeal cancer. Patients with tumors showing elevated COX‐2 expression might benefit from COX‐2 inhibitors, potentially in combination with other standard therapies like docetaxel.^175^ Targeting the Bromo‐ and Extra‐Terminal domain (BET) resulted in reduced stemness in HNSCC. BET inhibitors could be explored as potential therapeutic agents for patients with high CSC activity. Molecular profiling of tumors can help identify patients who may benefit from this type of treatment.^176^ To effectively utilize these findings in personalized HNSCC treatments, clinicians need to perform comprehensive molecular profiling of a patient's tumor. This can include assessing the expression levels of EGFR, IGF‐1R, STAT3, Wnt/β‐catenin, COX‐2, and other relevant biomarkers associated with CSC activity. Additionally, the identification of specific genetic mutations and alterations in the tumor can help guide treatment decisions.

### 4.3 Radiomics with AI and machine learning for HNSCC

In the past decade, there has been a surge in the adoption of artificial intelligence (AI) within the medical field.^177^ Its applications offer a promising avenue for error reduction, heightened efficiency, and unveiling critical disease insights for healthcare professionals.^178^, ^179^ Recent research has centered on leveraging machine learning (ML) to construct predictive models, identifying intricate data patterns that could revolutionize clinical decision‐making.^180^, ^181^ Prior studies have further indicated the potential of ML in optimizing workflow management specifically within radiation oncology.^182^

Radiomics, a method used to define tumor characteristics, involves extracting extensive data from clinical images.^183^ Inferences concerning tumor histology, grading, metabolism, and even patient survival are made possible by these quantitative features, which provide comprehensive insights into tumor heterogeneity, texture, intensity, and shape.^184^, ^185^ With noninvasive, quick, and affordable treatments, this quantitative image feature‐based technique could provide prognosis indications, which is a substantial development in clinical practice.^186^

Radiogenomic investigations have unveiled the genetic profiles underlying cancer, potentially offering additional prognostic insights. Typically, patients undergo invasive biopsies to determine tumor histology and oncological diagnosis.^187^ The tumor/node/metastasis (TNM) staging method, which is based on the tumor's resectability and grades, is frequently used to guide treatment options. Combining radiomics data with biological and prognostic information could reduce the need for invasive procedures.^186^ Aerts et al. demonstrated the clinical impact of radiomics in both HNSCC and non‐small cell lung cancer (NSCLC).^188^ They developed prediction models for survival utilizing diverse image biomarkers and the status of human papilloma virus‐16 (HPV‐16). Notably, these biomarkers within tumors and their heterogeneity were linked to radiation sensitivity or resistance. Numerous research studies examined the complex connection between radioresistance mechanisms and the molecular makeup of lung cancer. Others explored potential connections between genomic diversity and metastasis likelihood using MRI models.

ML initiatives have developed prediction models employing various techniques like penalized logistical regression, artificial neural networks (ANNs), Bayesian networks (BNs), decision trees (DTs), and support vector machines (SVMs). Studies have highlighted SVM's impressive accuracy in classifying survival and recurrence among patients with breast cancer, oral cancer, and cervical cancer.^189^, ^190^, ^191^, ^192^, ^193^, ^194^, ^195^ Segmentation plays a vital role in radiomics analysis, particularly in radiotherapy treatment.^196^ The planning target volume (PTV), which includes the gross tumor volume (GTV) and allows for daily setup uncertainties and organ movements during treatment, accounts for potential microscopic tumor spread. The GTV determines the location and size of the visible tumor. When planning radiotherapy, it is crucial to consider radiation doses to critical normal tissues (OARs) to preserve their function safely.^197^

**FIGURE 4:**
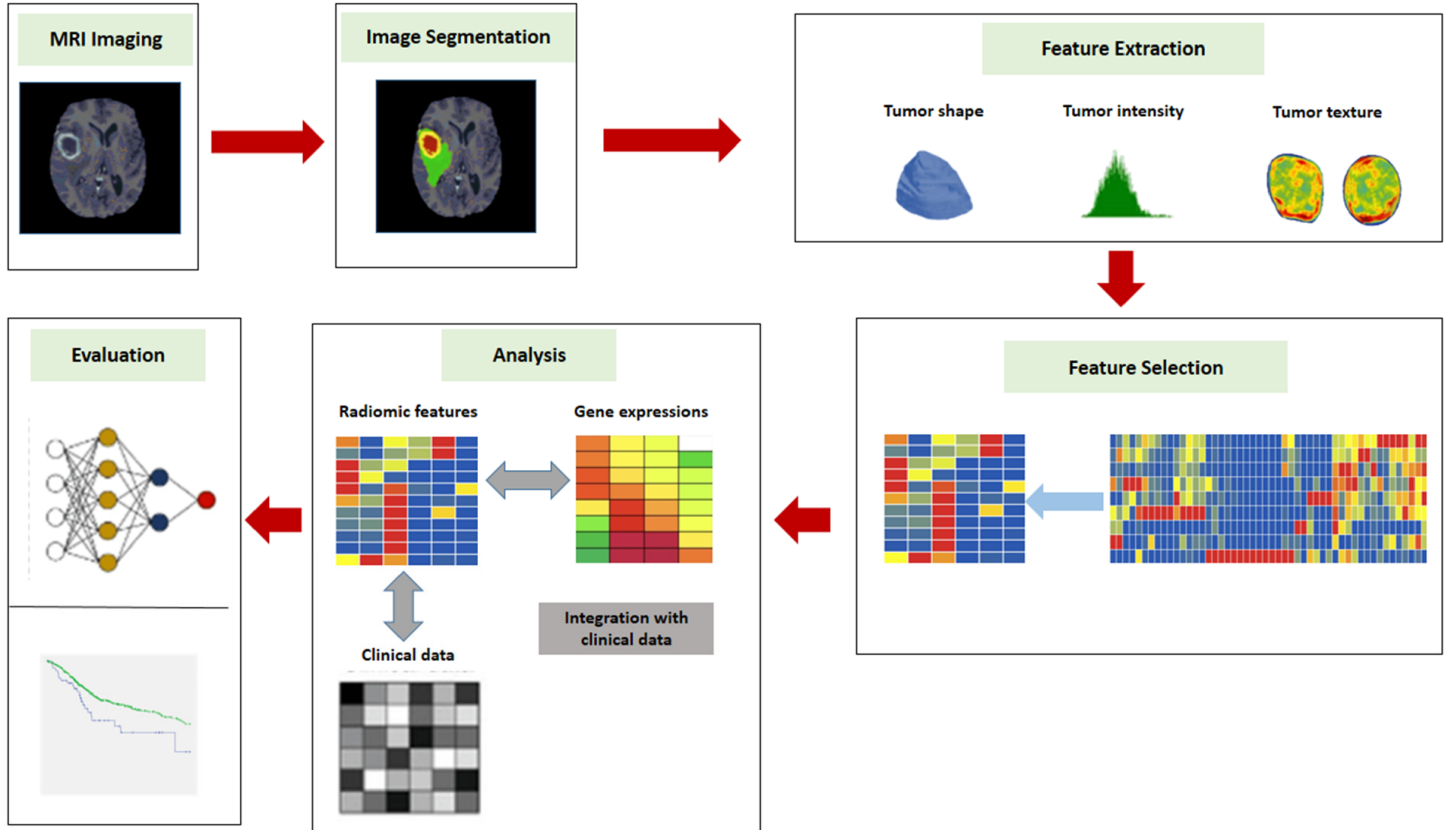
Typical radiomics workflow pipeline.

Balancing the delivery of a consistent high dose to GTV and PTV while minimizing OAR exposure is a pivotal concern in radiotherapy planning. Accurate delineation of GTV and PTV significantly influences the success of radiotherapy treatment, making them essential components in radiotherapy planning CT images. This study looks into how radiomics properties from PTV and GTV can be used to predict treatment outcomes, such as prognosis and recurrence rates, with accuracy. Figure 4 depicts the radiomics procedure in general.

#### 4.3.1 Application of radiomics and ML in HNSCC

Studies on radiomics in HNSCC have been published in a number of magazines recently. The diagnostic prediction of radiomics in HNSCC, including pathological subtypes, pre‐treatment staging, tumor differentiation from inflammation or necrosis, and tumor status prediction after treatments, including the presence or absence of specific pathogenic viruses, early recurrence or lymph node metastasis (LNM) prediction, survival prediction, and treatment‐related adverse reactions, are the main areas of focus for these studies.

##### Pre‐treatment related predictive modeling

Pre‐treatment staging stands as a critical facet in diagnosing tumors and holds strong connections to the prognosis of cancer patients. Research indicates that the T‐stage of head and neck tumors, along with viral‐relation status and involvement of lymph node, significantly affect patient prognoses. However, existing diagnostic techniques like serological testing, pre‐treatment tissue biopsy, and traditional medical imaging can only offer limited insights into tumor staging. These methods tend to be localized, qualitative, and subjective in their assessments. Leveraging radiomics for tumor staging before treatment offers a dependable and objective avenue. This approach can effectively guide treatment decisions, potentially reducing recurrence rates and minimizing adverse events.

Wang et al. created a T‐staging model for locally advanced laryngeal carcinoma (LC)^198^ by combining radiomics and ML techniques. The area under the receiver operating characteristic curve (AUC) was used to evaluate the model's performance. A nomogram that combined the radiomic signature and the T category determined by radiologists showed the best predictive ability, with an AUC of 0.892 (95% CI: 0.811–0.974).^196^ Ren et al. conducted a study involving 85 patients in a training cohort, utilizing MRI imaging features. Their findings showcased that the MRI radiomics signature successfully differentiated between stage III‐IV and stage I‐II HNSCC.^199^ For the purpose of forecasting the histologic grade of squamous cell carcinoma (SCC) in regions such as the oral tongue and tongue and floor of the mouth, radiomics offers a potentially useful non‐invasive method. Radiological analysis has been crucial in the development of non‐invasive biomarkers for HNSCC, successfully distinguishing well‐differentiated from moderately‐ and poorly‐ differentiated HNSCC with an accuracy of 0.96 and 0.92 AUC. https://doi.org/10.21873/anticanres.13949. Leveraging CT‐based radiomics models holds potential in predicting characteristics traditionally identified through pathologic assessment of HNSCC.^200^

In a prospective trial conducted using consecutive patients who had neck MR scans followed by thyroidectomies, it had been shown that ML‐based MRI models may differentiate between aggressive and non‐aggressive papillary thyroid cancer (PTC) prior to surgery. This aids in tailoring personalized treatment plans for PTC.^201^ These studies suggest that radiomics prediction models hold promise as an additional non‐invasive diagnostic tool for HNSCC pre‐treatment. This might result in more precise and objective tumor staging, and prediction of tumor malignancies, in order to guide subsequent treatment decisions.

In recent years, numerous studies have focused on predicting tumor responses to diverse treatments. While surgery remains a primary option for HNSCC, alternative treatments like induction chemotherapy, concurrent chemoradiation, targeted therapy, and immunotherapy exist. Establishing a sequential treatment strategy early on is crucial for devising personalized plans for cancer patients. Developing a radiomics model capable of predicting treatment outcomes or the likelihood of post‐treatment complications plays a pivotal role in achieving these goals.

Zhao et al., Wang et al., and Bologna et al. conducted retrospective analyses, extracting radiomics signatures from MRI images of NPC patients. They utilized the least absolute shrinkage and selection operator (LASSO) to identify pertinent radiomics features, constructing robust models to predetermine early reactions to induction chemotherapy in NPC patients. This approach aids in personalizing risk assessment and treatment stratification for individuals with NPC.^202^, ^203^, ^204^ Jin et al. presented preliminary findings based on radiomics features extracted from CT scans of 70 patients with esophageal cancer (EC). Their study showcased that combining radiomic features with dosimetric parameters resulted in a promising model. This combined model outperformed using radiomic features alone, specifically in predicting treatment responses among EC patients undergoing concurrent chemoradiation.^205^

Radiation therapy for HNSCC often leads to acute xerostomia, a prevalent side effect. Pota et al. and Liu et al. conducted studies on HNSCC and NPC respectively, utilizing CT scans taken before, during, and after treatment. They extracted imaging features to establish predictive models during initial treatment phases. These models aimed to forecast the onset of acute xerostomia in cancer patients following radiation treatment. Radiation‐induced brain damage is a frequent side effect in post‐radiation treatment for NPC, impacting patients' quality of life. In a study involving 242 NPC patients who underwent radiation therapy, researcher's analyzed MRI multiple‐weighted images, extracting 10 320 textural features. Using the RF method, they developed three prediction models capable of dynamically anticipating radiation‐induced brain injury.^206^ Because of these models, clinicians can diagnose injuries early and take preventive action to stop or decrease the injury's progression. These studies delved into the potential of radiomics in predicting HNSCC treatment responses or early identification of post‐treatment complications.

##### Models for survival, metastasis, and recurrence prediction

Extensive research in HNSCC has explored radiomics as a tool for prognostication, focusing on key indicators like OS, PFS, five‐year survival rate, DM, and local recurrence (LR). Despite advancements in therapeutic drugs, the challenge persists as many patients are identified in late stages due to the specific growth site of HNSCC.^207^, ^208^ Consequently, the prognosis remains weak, with the five‐year survival rate varying for hypopharyngeal cancer from 25% and upto 80% for NPC. Consequently, there is a strong interest among scientists to improve the accuracy of predicting LR, LNM, and even DM in HNSCC, along with enhancing the prediction of patients' survival rates.^209^ This pursuit aims to facilitate the development of more tailored and personalized treatment schemes for HNSCC patients.

Radiomics scores derived from the Cox proportional hazards regression model prove reliable in predicting local recurrence‐free survival (LRFS) for individuals with non‐metastatic T4 NPC, potentially offering guidance for personalized treatment strategies.^210^ Moreover, numerous studies continue to explore imaging genomics in combination with various ML algorithms to construct predictive models for HNSCC's LR. For instance, the M.D. Anderson Cancer Center's head and neck quantitative imaging working group examined CT/MRI and PET images from 465 HNSCC patients. Employing ML methods, they developed a radiomic signature comprised of distinct features with significant prognostic value, derived specifically from pre‐treatment imaging, resulting in two distinct radiographic signatures.^211^ Additionally, predictive radiomics models have found application in thyroid and EC research.^212^, ^213^, ^214^, ^215^, ^216^ Accurate prediction of LNM holds significant importance as a prognostic factor in HNSCC and is crucial for optimizing treatment strategies. Since a doctor's experience plays a major role in LNM identification accuracy, medical professionals stand to gain much from the development of automated LNM prediction models. Deep learning learns these features on its own; radiomics models typically rely on manually created features. Researchers have suggested hybrid prediction models that combine these strategies to improve forecast accuracy.^217^, ^218^, ^219^ In one study, utilizing the PyRadiomics platform, imaging features were extracted from primary tumors of 176 NPC patients without DM before treatment. Through LASSO algorithms and minimum redundancy‐maximum relevance, they identified robust features and constructed a logistic model for DM prediction.^220^

In these exploratory studies, researchers frequently used manual or semi‐manual methods to extract radiomics signatures from different kinds of imaging in training cohorts. They then employed ML algorithms to distill meaningful patterns and construct prediction models. Subsequently, independent cohorts were used to validate the models' efficacy. Table 2 provides an overview of representative studies showcasing these predictive models in HNSCC.

**TABLE 2:** Radiomics predicts recurrence and metastasis of head and neck cancer.

Study	Tumor type	Number of patients	Imaging modality	Machine learning algorithm	Outcome, feature selection model
Bahig et al.^221^	LHSCC	Total: 176 pts, 20 supraglottic, 5 pyriform sinus tumors	DECT	DT	LRR. Univariate Cox regression
Lu et al.^215^	PTC	Train: 154 pts, validation: 67 pts	Non‐ contrast and venous CE‐ CT	SVM, logistic regression	LNM. SVM. Multivariable logistic regression
Qu et al.^216^	EC	Train: 90 pts, validation: 91 pts	MRI	LASSO, elastic net regression, logistic regression	LNM. LASSO. Multivariable logistic regression
Zhang et al.^210^	NPC	Train: 80 pts, validation: 60 pts	T2, CE‐ T1	Logistic Regression	LR‐free survival. Radiomics score, Cox regression
Bogowicz et al.^222^	HNSCC	Train: 93 pts, validation: 56 pts	CE‐ CT	Logistic regression, PCA	LC and HPV status. PCA in combination with univariable logistic regression. Multivariable logistic regression
Tang et al.^223^	HNSCC	Train: 188 pts	CT	TCIA, DL‐ANN	GTV and PTV radiomics features
Martens et al.^224^	HNSCC	Train: 103 pts, validation: 71 pts	PET, low‐dose‐CT	Logistic regression	LR, DM, OS. RadCat tool Cox regression analysis. Multivariable logistic regression
Li et al.^217^	NPC	Total: 306 pts, 20 of whom developed with recurrence	CT, MR, PET	PCA, ANN, KNN, SVM	LR. PCA. Machine learning classifiers
Liu et al.^215^	PTC	Total: 120 pts	Preoperative ultrasound images	SVM	Metastasis. Support vector machine classifier
Wu et al.^219^	HNSCC	Train: 141 pts, validation: 96 pts	PET, CT	PCA	LR. PCA. Multivariate Cox proportional hazards regression
Zhou et al.^218^	HNSCC	Total: 188 pts	PET, CT	SVM, DT and KNN	DM. Machine learning classifiers
Bogowicz et al.^225^	HNSCC	Train: 128 pts, validation: 50 pts	PET, CT	PCA, LASSO	LR. PCA and LASSO. Multivariable Cox regression
Tan et al.^212^	ESCC	Train: 154 pts, validation: 76 pts	Arterial‐ phase CT	LASSO, logistic regression	LMR. Rad‐score, logistic regression
Vallieres et al.^226^	HNSCC	Total: 300 pts	Pre‐ treatment FDG‐PET and CT	Random forests	LR and DM. Machine learning classifier
Kwan et al.^227^	HPV‐related Oropharyngeal Carcinoma	Train: 300 pts, validation: 36 DM pts	CT	Logistic regression	DM. PyRadiomic. Radiomics score
Park et al.^213^	PTC	Train: 400 pts, validation: 368 pts	Neck ultrasound	LASSO	LNM. LASSO. Rad‐score, LASSO regression
Zhang et al.^228^	Non‐metastatic T4 NPC	Train: 360 pts, validation: 120 pts	T1, T2, CE‐ T1	Logistic regression	LR, Rad‐score, cox proportional hazards regression
Zhang et al.^220^	NPC	Total: 176 pts	PET, CT	Logistic regressin	DM. LASSO. Multivariate logistic regression
M.D. Anderson Cancer^211^	HNSCC	Train: 255 pts, tune: 165 pts, validation: 45 pts	CT, MRI, PET	DT	5‐year LCR. Multivariable Cox regression
Martens et al.^224^	HNSCC	Train: 103 pts, validation: 71 pts	18F‐FDG‐ PET, CT	Logistic regression	LR, DM, OS. Rad‐ score. Multivariable survival regression

Research reports on the application of radiomics in HNSCC predominantly focus on survival prediction models. For instance, Shen et al. investigated the predictive value of an MRI‐based radiomic model for PFS in nonmetastatic NPC. Analyzing clinical and MRI data from 327 NPC patients, they established five models. Evaluation using Harrell's concordance index (C‐index) revealed that a model combining radiomics, overall stage, and EBV DNA exhibited superior performance in predicting PFS for nonmetastatic NPC patients.^229^

In HNSCC, Yuan et al. used a training cohort of 85 patients and employed LASSO Cox regression to select pertinent prognostic features, generating an MRI‐based radiomic signature. Their findings highlighted that this radiomic signature serves as an independent prognostic factor for HNSCC patients' outcomes.^230^ Other studies have identified robust machine‐learning methods for OS prediction in head and neck cancer patients. Moreover, the utilization of pre‐ and post‐operative PET/CT radiomics features in HNSCC demonstrated improved predictions for OS and disease‐free survival (DFS) when combined with clinicopathological characteristics.^231^ The range of survival predictions across different types of HNSCC using radiomics signature models derived from various imaging sequences is summarized. This includes specific survival values predicted by each model and the ML algorithms utilized for these predictions.

##### Other predictive models

Tumor heterogeneity stands as a significant prognostic factor in HNSCC. Conventional tumor markers obtained from tissue and blood frequently do not have the spatial resolution required to capture this heterogeneity. HNSCC growth sites are hidden, making biopsies both before and after treatment difficult to obtain. Furthermore, the markers obtained from these biopsies frequently only reflect a portion of the tumor at a certain moment in time, which restricts their capacity to reflect the biology of the tumor or biological alterations that occur both during and after therapy. Gu et al. demonstrated the remarkable predictive ability of a radiomics model based on CT images for identifying the presence of specific markers like cytokeratin 19, galectin 3, and thyro‐peroxidase, aiding in distinguishing benign and malignant thyroid nodules.^232^ Chen et al. delved into the correlation between programmed cell death protein 1 ligand (PD‐L1) immunohistochemical expression and PET/CT radiomics. Their findings indicated that several PET/CT‐derived textural features, in combination with p16 and Ki‐67 staining percentages, could offer supplementary information to identify tumor PD‐L1 expression in HNSCC.^233^ Researchers have also been concentrating on the correlation between radiomics aspects and the molecular properties of HNSCC in recent years. Additionally, there is a rising interest in evaluating how well radiomics models that are derived from several imaging modalities predict the same disease within a given environment.^234^

#### 4.3.2 Future directions, challenges, and barriers of radiomics

The advancement of radiomic analysis hinges on the development of practical decision support and prognostic tools for routine clinical use. However, certain hurdles in quantitative imaging need addressing:

Despite promising results in exploratory radiomics studies, there is a lack of extensive independent validation. A recent study by Kim et al. reviewed 516 AI algorithm‐based diagnostic analyses of medical images. Shockingly, only 31 studies (6%) validated their models in external test cohorts, which encompass data from institutions different from the one providing the training data or from the same institution but from a distinct time period. The reliance on homogeneous, single‐institution, or single‐scanner training data could restrict the applicability of radiomics‐based models. This emphasizes the necessity for multi‐institutional and multinational medical imaging databases to develop robust radiomics tools for clinical use. Data sharing is pivotal in overcoming the scarcity of diverse imaging data.^235^ Platforms like “The Cancer Imaging Archive” (TCIA) have been established to facilitate this, offering de‐identified imaging collections alongside clinical data and digital infrastructure for data sharing. As of December 2019, TCIA has nine HNSCC collections featuring CT, MRI, and FDG‐PET imaging data.

Imaging data variability can be partially reduced by applying preprocessing techniques like resampling and filtering (Figure 4). But as the industry moves closer to the clinical use of AI‐driven image analysis, standardization of reconstruction and acquisition parameters between providers and manufacturers becomes imperative.

Variability in delineating volumes of interest (VOI) or regions of interest (ROI) among different observers or even within the same observer can be mitigated using semi‐automated or automated segmentation tools. Efforts to standardize radiomics features have been undertaken, notably through initiatives like “The image biomarker standardization initiative” (IBSI). Additionally, the use of open‐source feature libraries and radiomics extraction software packages such as “PyRadiomics” or the “Imaging Biomarker Explorer” facilitates reproducible feature extraction. These tools also aid in easy reporting of radiomics feature definitions and are increasingly being adopted in recent publications.

## 5 FUTURE DIRECTION

In future, personalized HNSCC medicine is expected to become even more prevalent as advances in technology and medicine, like PM, genetic testing, molecular biomarkers, AI, gene editing, stem cell therapies, and so forth continue to deepen our knowledge of the genetic and molecular alterations which drive the development and spread of cancers. For example, genetic testing is becoming increasingly common, allowing doctors to identify specific genetic mutations that may drive the growth of particular cancer. Targeted therapies can then be created using this knowledge that are more likely to be effective at killing cancer cells while minimizing the side effects experienced by patients.

The treatment of advanced or recurrent cancer is one area where personalized cancer medicine is anticipated to have a big impact by identifying genetic and environmental risk factors for cancer, healthcare providers can develop personalized prevention and management plans customized to the requirements of each individual patient. This may involve lifestyle changes, such as diet and exercise, as well as medications and other therapies. A potential challenge to the widespread adoption of personalized cancer medicine is the cost. Many of the technologies and techniques used in cancer personalized medicine, such as genetic testing and targeted therapies, can be expensive. This may limit their availability to those who can afford them, leading to a potential inequality in access to care. However, as these technologies and techniques become more widespread and cost‐effective, it is likely that the benefits of cancer‐personalized medicine will become more widely available to a larger number of people.

## 6 CONCLUSION

Personalized cancer medicine has the potential to revolutionize the way we think about cancer care and improve the lives of cancer patients around the world. Tailoring cancer treatment to the unique characteristics of each personalized cancer medicine can provide more effective and targeted therapies, particularly for advanced or recurrent cancer. While there may be challenges to its widespread adoption, due to its higher costs, it is expected that personalized cancer medicine will continue to grow and evolve in the future, bringing significant benefits to cancer patients and healthcare providers alike.

## AUTHOR CONTRIBUTIONS

**Shalindu Malshan Jayawickrama:** Conceptualization (lead); data curation (lead); formal analysis (lead); funding acquisition (lead); investigation (lead); methodology (lead); project administration (equal); resources (lead); software (lead); validation (lead); visualization (lead); writing – original draft (lead); writing – review and editing (lead). **Piyumi Madhushani Ranaweera:** Data curation (supporting); formal analysis (supporting); investigation (supporting); methodology (supporting); project administration (equal); resources (lead); software (lead); validation (lead); visualization (lead); writing – original draft (lead); writing – review and editing (lead). **Ratupaskatiye Gedara Gunaratnege Roshan Pradeep:** Data curation (equal); investigation (equal); methodology (equal); resources (equal); software (equal); visualization (equal); writing – original draft (equal); writing – review and editing (lead). **Yovanthi Anurangi Jayasinghe:** Resources (equal); writing – review and editing (equal). **Kalpani Senevirathna:** Resources (equal); validation (equal); visualization (equal); writing – review and editing (equal). **Abdul Jabbar Hilmi:** Resources (equal); writing – review and editing (equal). **Rajapakse Mudiyanselage Gamini Rajapakse:** Resources (equal); supervision (supporting); writing – review and editing (equal). **Kehinde Kazeem Kanmodi:** Funding acquisition (equal); resources (equal); writing – review and editing (supporting). **Ruwan Duminda Jayasinghe:** Funding acquisition (equal); resources (equal); supervision (lead); writing – review and editing (lead).

## FUNDING INFORMATION

This study was self‐funded.

## CONFLICT OF INTEREST STATEMENT

The authors have stated explicitly that there are no conflicts of interest in connection with this article.

## ETHICS STATEMENT

Not applicable. This study did not collect data from human or animal subjects but an open research repository.

## Data Availability

Data sharing is not applicable to this article as no new data were created or analyzed in this study.
